# Physical and Mechanical Properties of a Bulk Lightweight Concrete with Expanded Polystyrene (EPS) Beads and Soft Marine Clay

**DOI:** 10.3390/ma12101662

**Published:** 2019-05-22

**Authors:** Jianguo Wang, Bowen Hu, Jia Hwei Soon

**Affiliations:** 1 School of Mechanics and Civil Engineering, China University of Mining and Technology, Xuzhou 221116, China; bowenhu123@163.com; 2 State Key Laboratory for Geomechanics & Deep Underground Engineering, China University of Mining and Technology, Xuzhou 221116, China; 3 Center for Offshore Research and Engineering, National University of Singapore, E1A-07-03, 1 Engineering Drive 2, Singapore 117576, Singapore; jgwangau@gmail.com

**Keywords:** lightweight concrete, soft marine clay, expanded polystyrene beads, stress–strain behaviors, failure pattern, compressive strength

## Abstract

The variation of physical and mechanical properties of the lightweight bulk filling material with cement and expanded polystyrene (EPS) beads contents under different confining pressures is important to construction and geotechnical applications. In this study, a lightweight bulk filling material was firstly fabricated with Singapore marine clay, ordinary Portland cement and EPS. Then, the influences of EPS beads content, cement content, curing time and confining pressure on the mass density, stress–strain behavior and compressive strength of this lightweight bulk filling material were investigated by unconsolidated and undrained (UU) triaxial tests. In these tests, the mass ratios of EPS beads to dry clay (E/S) were 0%, 0.5%, 1%, 2%, and 4% and the mass ratios of cement to dry clay (C/S) were 10% and 15%. Thirdly, a series of UU triaxial tests were performed at a confining pressure of 0 kPa, 50 kPa, 100 kPa, and 150 kPa after three curing days, seven curing days, and 28 curing days. The results show that the mass density of this lightweight bulk filling material was mainly controlled by the E/S ratio. Its mass density decreased by 55.6% for the C/S ratio 10% and 54.9% for the C/S ratio 15% when the E/S ratio increased from 0% to 4% after three curing days. Shear failure more easily occurred in the specimens with higher cement content and lower confining pressure. The relationships between compressive strength and mass density or failure strain could be quantified by the power function. Increasing cement content and reducing EPS beads content will increase mass density and compressive strength of this lightweight bulk filling material. The compressive strength with curing time can be expressed by a logarithmic function with fitting correlation coefficient ranging from 0.83 to 0.97 for five confining pressures. These empirical formulae will be useful for the estimation of physical and mechanical properties of lightweight concretes in engineering application.

## 1. Introduction

A large amount of soft marine clays have been excavated from civil engineering projects in coastal areas. These excavated soft marine clays are not suitable directly as construction materials due to high water content, high compressibility, low bearing capacity, low stiffness, low permeability and low shear strength [1,2,3,4]. However, these clays can be used as sustainable building materials after their mechanical properties are modified by Portland cement or other cementitious materials [5,6,7,8,9,10,11]. The mixture of air foam, natural clay and cement is called “lightweight cement clay” or “air cement mixed clay”. Lightweight cement clay has been widely used in transportation infrastructures such as embankments construction, airport, canal linings, bridges construction and underground engineering [12,13,14,15,16,17]. Thus, how to use these soft marine clays is related to an environmental problem for the sustainable development in civil engineering.

Lightweight cement clay materials have attracted more and more attentions in civil engineering. Horpibulsuk et al. [18] reported the process of manufacturing lightweight cemented clay. Their process is as follows: Water is firstly added to the clay to obtain clay muddy paste. The clay muddy paste is mixed with the Portland cement in a mixing chamber. The cement clay mixture is then transferred to the air foam mixing plant and mixed with air foam to obtain the lightweight cement clay with high workability and low density. The air foam increases pore space and reduces the unit weight and strength of this soft clay.

Expanded polystyrene (EPS) beads have been widely used as aggregates of building materials in the construction of high-rise buildings and long-span bridges where the self-weight of structure member has become an important load [19,20]. Lee et al. [21] investigated the composite sandwich plate of ultra-high performance concrete and EPS beads. They also explored the potential application of such sandwich plates in high-rise buildings. In addition, EPS beads are of low density and high compressibility. They are often used as filling materials in earthquake buffers, such as backfilling materials for retaining walls, and filling materials for trenches [21,22,23,24]. These lightweight filling materials can be used as a buffer layer to reduce the dynamic earth loads for the seismic effect of rigid basements and retaining walls. Bathurst and Zarnani [23] and Gao et al. [25] conducted a series of shaking table tests to study the seismic performance of the EPS block geofoams. They found that the EPS can effectively reduce the seismic load and the lateral thrust increment of the rigid basement and retaining wall.

Recycling EPS as building materials can meet the requirements of economic and environmental protection [26] because EPS beads are difficultly degraded in a natural way. Fernando et al. [27] explored the use of mechanical recycling of EPS beads to make durable lightweight panels as wall materials for buildings and houses. These panels can be fabricated quickly and easily and used as good wall decorations without plaster, thus being a benefit to the environment. EPS beads have the advantages of low density, hydrophobicity and heat insulation. These can meet the usage requirements for heat insulation and lightweight [28,29,30]. Thus, the design and fabrication of this lightweight concrete (cement soil) for expected mechanical properties is a necessary topic.

The physical and mechanical properties of lightweight cement materials have been investigated at various cement contents and curing times [5,10,13,31,32,33,34,35,36]. These properties include density, hydraulic conductivity, compressive strength, stiffness, stress–strain behaviors and the dissipation phenomena. Giorgio and Scerrato [35] observed the dissipation phenomena under uniaxial compressive tests and proposed a micro-nonlinear 3D model to describe the dissipation phenomena in concrete. Horpibulsuk et al. [12,13,18] proposed a key parameter of void/cement V/C, which is the ratio of the volume of void to the volume of cement. The parameter V/C can reflect the comprehensive influence of cement content, air content and water content on the stress–strain behaviors and strength. Tsuchida and Tang [5] proposed a new formula to estimate the strength of lightweight cement clay. Their formula was validated by the test data of compressive strength of six lightweight cement clays with different initial water content. Hu et al. [37] investigated the mechanical behaviors of soft clay under complex cyclic stress paths. They found that the cyclic strength, cyclic modulus and cyclic strain of soft clay are significantly correlated with bidirectional shear frequency and cyclic shear stress ratio. Placidi et al. [38] provided an explicit evolution of damage field with loading and discussed a novel dependence of the stiffness coefficients on the damage field. Recently, in order to save cost and protect environment, some industrial or agricultural wastes such as EPS beads [39,40], fly ash (FA) [9,33], biomass ash (BA) [2], rice husk ash [10,39], reactive MgO [11,15,41] and rubber aggregates [42] are mixed into the lightweight cement clay as bulk filling materials for embankments construction, airport, canal linings, bridges construction and underground coal mine [41,43]. For example, Wang et al. [11] investigated the compaction, mechanical and microstructural characteristics of reactive MgO lightweight soil with varying water-soil ratios, carbonation time and MgO-soil ratios. Cheng et al. [9] performed the isotropic consolidated drained triaxial tests of the fly-ash blended cement (FAC)-admixed marine clay under the confining pressure of 50 kPa to 350 kPa. Jamsawang et al. [44] investigated the effect of fiber types on the flexural performance of the cement-fiber-sand made from cement, sand, fibers and water. Fantilli and Chiaia [42] investigated the effect of rubber aggregates on the mechanical performances of the rubber concrete by the three point bending test. Therefore, the effect of each component on the physical and mechanical properties of lightweight cement clay is the focus.

The physical and mechanical properties of the EPS lightweight clay are important to successful applications in construction and geotechnical engineering. The mechanical properties of EPS lightweight clay vary with clay property, EPS property, cement content and their mass ratios. Yoonz et al. [45] tested the physical and mechanical properties of EPS lightweight soil by unconfined and triaxial compression tests and further analyzed the effects of initial water content, cement ratio, EPS ratio and curing pressure on the compressive strength of lightweight clay. Liu et al. [46] fabricated a new lightweight filling material by mixing polystyrene pre-puff (PSPP) beads with a China soft silty clay, cement and water. They found that the PSPP beads and cement are the most effective factor affecting the mass density and unconfined compression strength of EPS lightweight clay. Sadrmomtazi et al. [39] investigated the feasibility of multi-strength lightweight concrete containing expanded polyethylene beads. They used different proportions of EPS beads as a portion of aggregate replacement to reduce the weight of concrete. They have produced lightweight concrete of structure with medium strength and thermal insulation property. Liu and Chen [19] studied the influence of EPS beads size on the mechanical properties of EPS lightweight concrete. Their results show that the mechanical properties of EPS concrete are closely related to the size and content of EPS beads. Allahverdi et al. [20] produced a multi-strength green light active powder concrete with EPS beads as light aggregates to reduce the static load of concrete structures exposed to earthquake. They tried a new design and construction scheme for tall construction projects and long span bridges. Chung et al. [47] illustrated the influence of the size and arrangement mode of EPS beads on the performance of lightweight concrete. They concluded that the size or aggregation degree of polystyrene aggregates inside the concrete had significant influences on the performance of concrete. These physical and mechanical properties of each component can be used to control and develop the material properties of a high-performance EPS concrete. Previous studies paid more attention to developing novel cementitious materials, such as fly ash, reactive MgO and so on, which were used to improve the physical and mechanical characteristics of soft clay. However, few literatures have reported the changes of deformation and strength of lightweight cement clay vary with cement and EPS beads contents by the UU triaxial tests. The lightweight cement soft clay made from EPS beads and Singapore marine clay has not been investigated so far.

This study systematically investigated the stress–strain behaviors and compressive strength of lightweight cement clay with UU triaxial compression tests. Firstly, the effects of the mass ratios of EPS to clay and cement to clay on the mass density of lightweight cement clay after three curing days were analyzed. Then, the stress–strain behaviors of lightweight cement clay were studied in detail under different confining pressures, EPS to clay and cement to clay ratios after seven curing days. Third, the relationships between compressive strength and failure strain, mass density and curing time were expressed by a fitting formula. These empirical formulae have high correlation coefficients and can provide an effective engineering tool for predicting the strength of lightweight cement clay in engineering applications.

## 2. Materials and Methods

### 2.1. Materials

**Figure 1:**
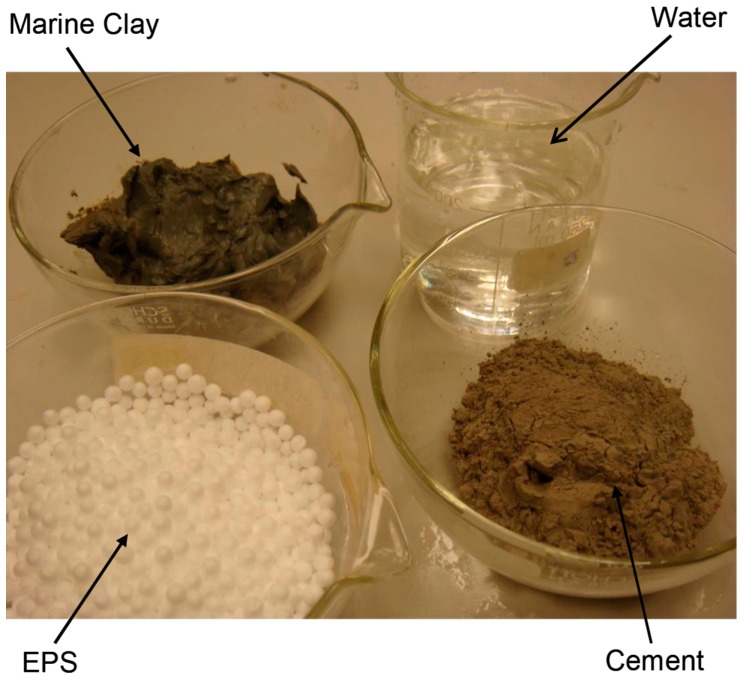
Four components for preparation of expanded polystyrene (EPS)-cement lightweight clays.

The lightweight cement clay in this study was composed of four components: Singapore marine clay, EPS beads, cement and water. They are presented in Figure 1. The marine clay was obtained from a construction site in Singapore. The physical properties and shear strength of this Singapore marine clay are listed in Table 1. The original water content, initial void ratio, specific gravity and bulk density were 54.19%, 1.29, 2.65 and 1.61 g/cm^3^, respectively. The liquid limit was 87.75% and the plastic limit was 40.04%, thus the plasticity index was 47.71%. The shear strength of this marine clay ranged from 18.6 kPa to 24.5 kPa, which indicates that the Singapore marine clay belongs to soft clay.

**Figure 2:**
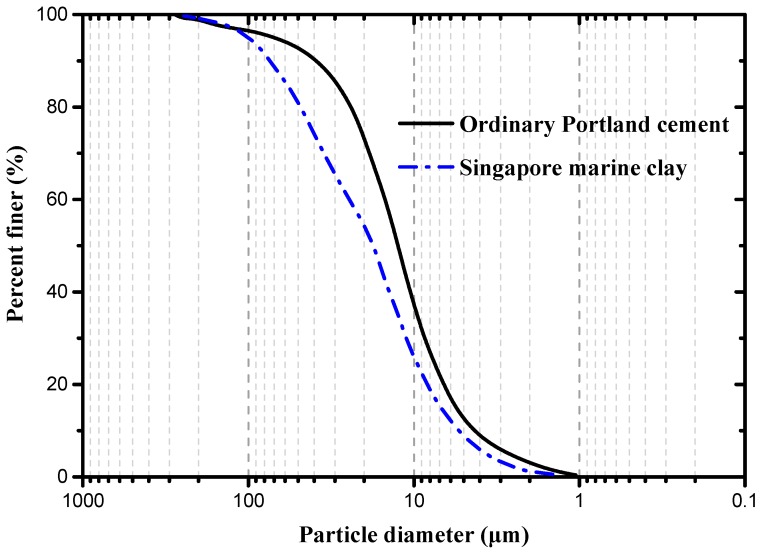
Particle size distribution of ordinary Portland cement and Singapore marine clay (the particle size distribution data are from Du and Pang [49]).

**Figure 3:**
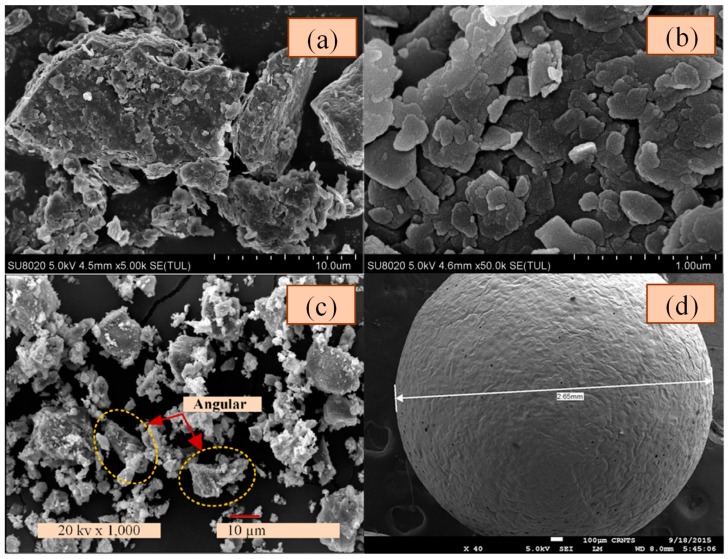
SEM images of four components: (**a**) Large particles of Singapore marine clay [49]; (**b**) small particles of Singapore marine clay [49]; (**c**) ordinary Portland cement (OPC) particles [2] and (**d**) an EPS bead [40].

The particle size distributions of ordinary Portland cement and Singapore marine clay are shown in Figure 2. The D_50_ (the diameter at which 50% of the particles has a smaller diameter) of marine clay and cement was 17.6 μm and 12.5 μm, respectively. Therefore, the average particle size of this marine clay was larger than the cement. The microstructure of marine clay can be observed by the scanning electron microscope (SEM) (FEI, Hillsboro, USA) analysis. The SEM images of Singapore marine clay microstructure are shown in Figure 3a,b. As shown in Figure 3a, there were some large particles in the Singapore marine clay and those large particles might be fine sand grains while the finer particles around them could be silt and fine clay particles. Much smaller particles of marine clay in the shape of irregular flakes are shown in Figure 3b. The microstructure of Singapore marine clay is relatively loose and the clay particles are assembled in a dispersed arrangement [48]. The majority of the marine clay is silica, and the content of SiO_2_ is as high as 75.42% in Singapore marine clay [49]. Based on the size distribution analysis of Singapore marine clay, Du and Pang [49] reported that the marine clay has a higher fraction of coarse grains due to higher content of sand. Ordinary Portland cement (OPC) was used to enhance the strength of cemented soft clay. The microstructure of the OPC particles is shown in Figure 3c, and the morphology of the OPC particles is angular and irregular. It can be also clearly seen that the particle size of marine clay was larger than cement from Figure 3a,c. This was also confirmed by Figure 2. The EPS beads were highly compressible, with a density between 13 kg/m^3^ and 15 kg/m^3^. Figure 3d shows the SEM image of an EPS bead [40]. The bead is a round, spherical shape with a rough surface, and a diameter between 2 mm and 5 mm. The EPS beads used in this experiment were ultralight, enclosed, rigid and plastic foam and were most commonly used as filling materials in building construction and geotechnical engineering.

### 2.2. Experimental Procedure

#### 2.2.1. Mixing Ratios and Preparation of Specimen

The mixing ratio is defined as the mass ratio of two components. The mass ratio has the ratio of water to dry clay (W/S ratio), the ratio of cement to dry clay (C/S ratio) and the ratio of expanded polystyrene to dry clay (E/S ratio). The W/S ratio is only set at 100%. The C/S ratio is taken as 10% and 15%, respectively. The E/S ratio is only 0.5%–4% and all mixing ratios used in this study are listed in Table 2.

At the sample preparation stage, water is added in the mixing process to enhance the workability of clay cake and to activate the hydration of cement. The cement is used as a solidification agent to enhance the strength of lightweight clay. When all materials are ready, the Singapore marine clay is placed into a concrete mixer and half of the added water according to the specified ratio is poured into the clay cake. Marine clay and water are mixed for 5–10 minutes to make homogenous slurry. Then, EPS beads are slowly added into the clay slurry and the mixing process is continued until the beads are evenly distributed within the slurry. Third, cement is added into the other half of the added water and poured into the clay slurry for mixing. This mixing process lasts for additional five minutes. The fresh EPS beads mixed lightweight clay is obtained in a slurry form. The overall mixing process takes around 15 minutes to 20 minutes.

**Figure 4:**
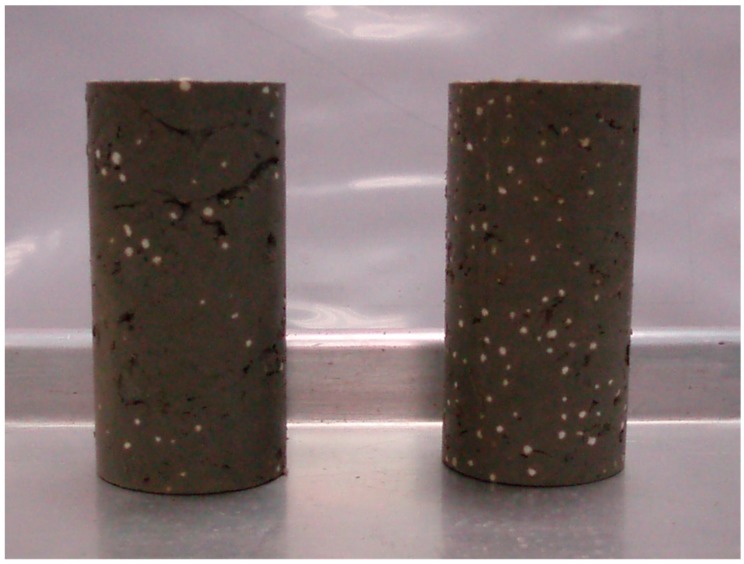
Prepared soil samples for Singapore marine clay (EPS: 1% for left and 2% for right).

After a thorough mixing, the well-mixed EPS lightweight clay is cast into specimens for the UU triaxial tests. A PVC mold is used to cast specimens with 100 mm in height and 50 mm in diameter. Figure 4 shows the prepared specimens when the ratio of EPS particles to clay (by weight) is about 1% (the left one) and 2% (the right one), respectively. The prepared specimens are placed into a plastic container and covered with wet cloths to prevent moisture loss until testing. The curing process of specimens is kept in a room temperature of 20 ± 2 °C. Finally, all specimens are being removed from the PVC mold on the third curing day and are ready for tests at the third curing day, seventh curing day and 28^th^ curing day.

#### 2.2.2. Test Method

**Figure 5:**
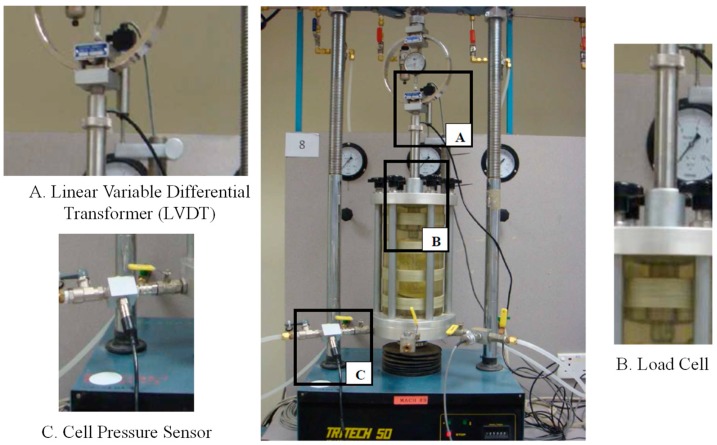
Unconsolidated and undrained (UU) triaxial test setup in laboratory.

After the specified curing days, the average diameter and height of each specimen were measured using a vernier caliper. These dimensions were used to determine the volume of each specimen. The mass of the specimen was weighted using an electric weighing machine (Sartorius, Göttingen, Germany). The density of the specimen was calculated based on these measurements. The EPS-cement mixed lightweight clay cannot be saturated through back pressure or soaking in water for long time. Back pressure may damage the microstructure and lose the truth of its mechanical properties. Therefore, the specimens were used for UU triaxial tests according to the test procedure in section 7 of BS1377. The deformation rate of 1.25 mm/min was applied until sample failure. This test can measure the mechanical properties of the lightweight clay such as Young’s modulus and shear strength. The compressive strength $q_{u}$ of UU triaxial tests was determined by the peak stress or the stress under a 20% axial strain, whichever is obtained first. In this study, the UU triaxial tests were conducted under confining pressures of 0 kPa, 50 kPa, 100 kPa and 150 kPa, respectively. In order to ensure the repeatability and reliability of test results, at least two tests were performed for each case. A typical test system is shown in Figure 5. Linear variable differential transformer (LVDT) (Inelta, Ottobrunn, Germany), a displacement measuring instrument, was used to measure the displacement of the specimen. This LVDT has the maximum displacement capacity of 25 mm. A load cell was used to measure the total load applied on specimen during the UU triaxial tests. A cell pressure sensor was installed and linked to the data logger to control the desired confining pressure. Before tests, this cell pressure sensor was calibrated to ensure no leakage around the sensor.

## 3. Results and Discussions

### 3.1. Mass Density

**Figure 6:**
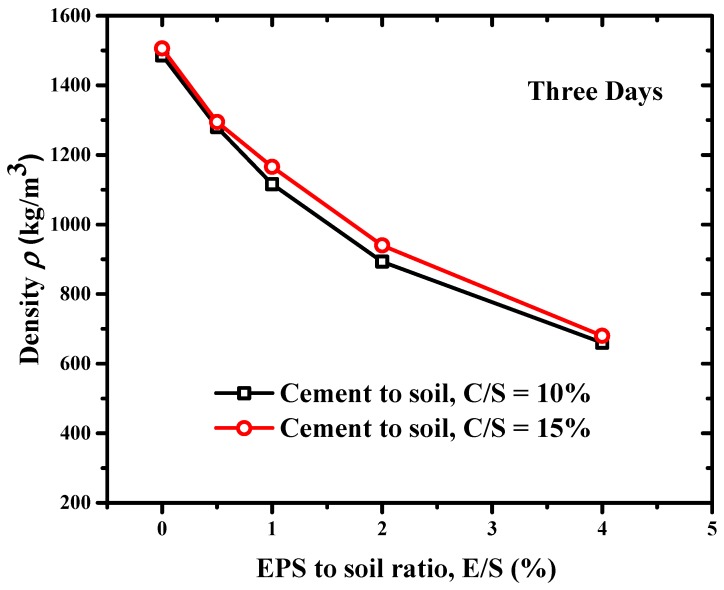
Effect of EPS to clay (E/S) and cement to clay (C/S) ratios on the density of EPS-cement lightweight clay.

The mass density and strength of mixed lightweight clay are key parameters to its applications in building construction and geotechnical engineering. The specimen after three curing days was taken out for measuring its (bulk) density. The mass was weighed and the diameters along the upper, middle and lower parts as well as the height were measured by a vernier caliper. The volume of specimen was calculated based on an assumption of a cylindrical sample, and the mass density was then calculated by the mass weight divided by the volume. The effects of E/S and C/S ratios on the mass density of mixed clay specimens after three curing days are shown in Figure 6. Increasing the C/S ratio of specimens could make the mass density increase slightly. Compared with cement, the EPS beads content had much more significant effects on the mass density of specimen. For the particular C/S ratio of 10%, the mass density of specimen was 1486 kg/m^3^ when the E/S ratio was zero while the mass density of specimen was only 660 kg/m^3^ when the E/S ratio was 4%. For the particular C/S ratio of 15%, the mass density of specimen was 1507 kg/m^3^ when the E/S ratio was zero while the mass density of the specimen was only 680 kg/m^3^ when the E/S ratio was 4%. The E/S ratio increased from 0% to 4% but the mass density of specimen decreased by 55.6% for the C/S ratio 10% and 54.9% for the C/S ratio 15%, respectively. This is because the EPS beads had a much lower unit weight but much bigger volume. The weight ratio of EPS beads to clay (E/S) was only 0.5–4% from the perspective of the clay mass, but the volume ratio of EPS beads to clay was 73% to 582% from the perspective of clay volume. Lower EPS beads and higher cement content mean much higher mass density of lightweight clay. Therefore, the E/S ratio of lightweight clay was a key parameter to control the mass density of lightweight clay.

### 3.2. Stress–Strain Behaviors

**Figure 7:**
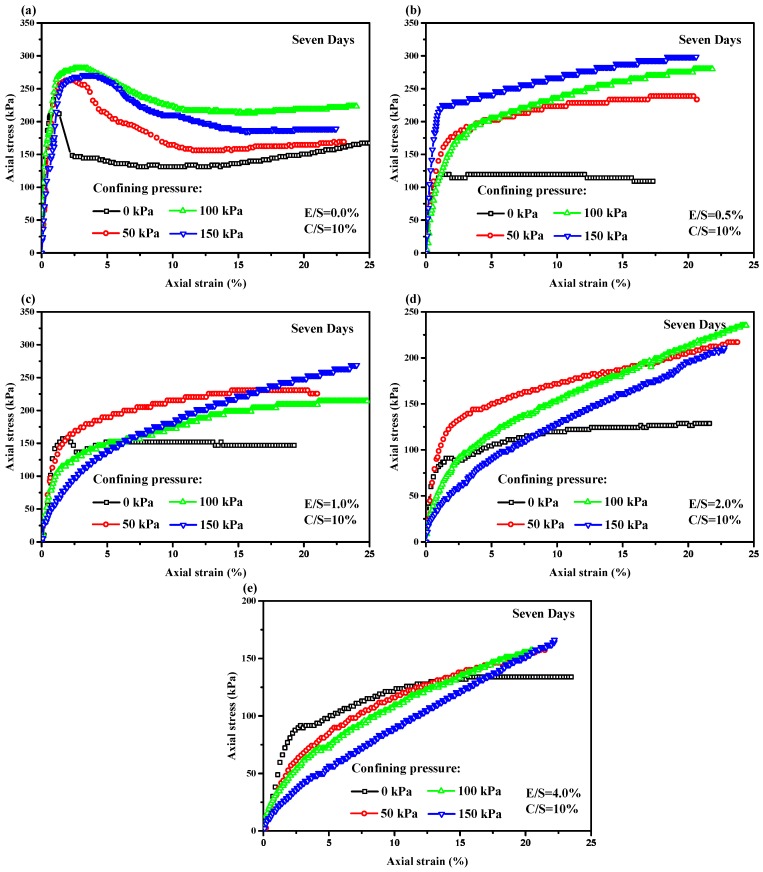
Axial stress and axial strain curves of EPS-cement lightweight clay with different confining pressures for seven days curing period with cement ratio 10% for all EPS ratios (**a**) 0%; (**b**) 0.5%; (**c**) 1.0%; (**d**) 2.0% and (**e**) 4.0%.

**Figure 8:**
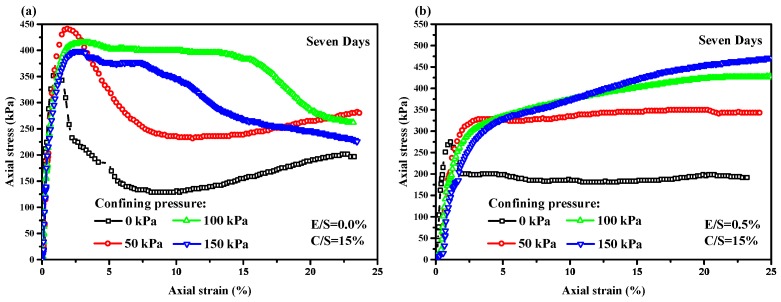

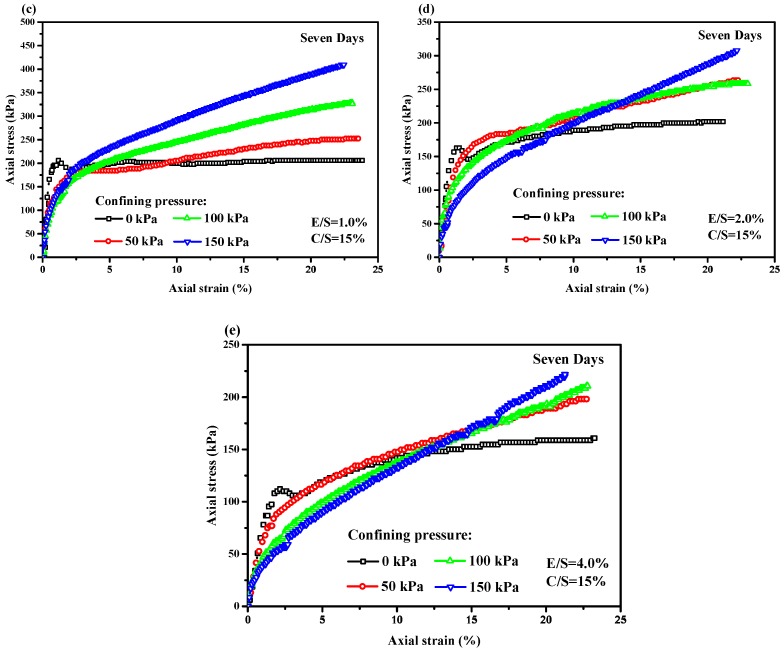
Axial stress and axial strain curves of EPS-cement lightweight clay with different confining pressures for seven days curing period with cement ratio 15% for all EPS ratios (**a**) 0%; (**b**) 0.5%; (**c**) 1.0%; (**d**) 2.0% and (**e**) 4.0%.

**Figure 9:**
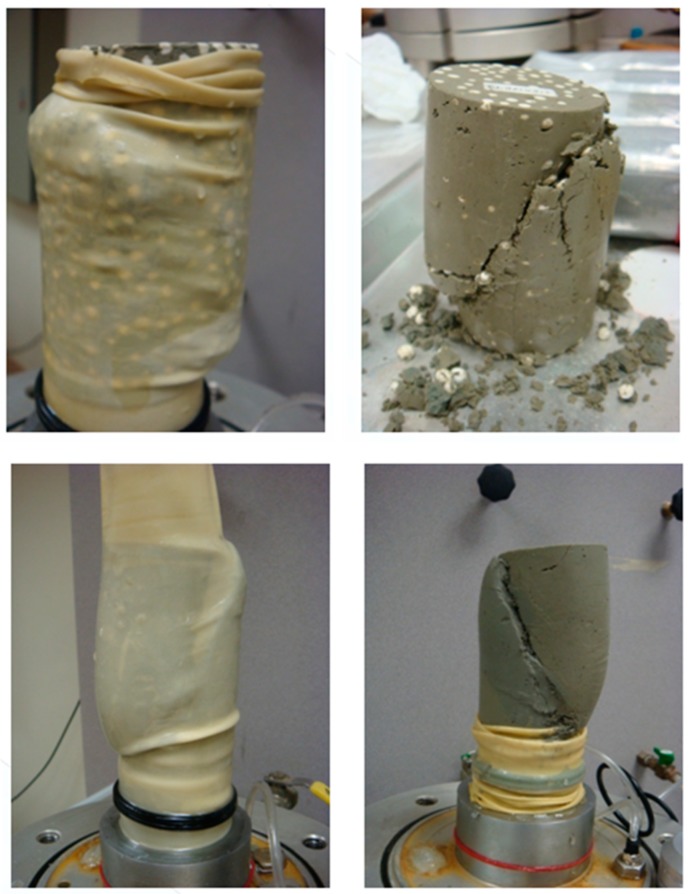
Shear failure for the lower E/S ratio.

**Figure 10:**
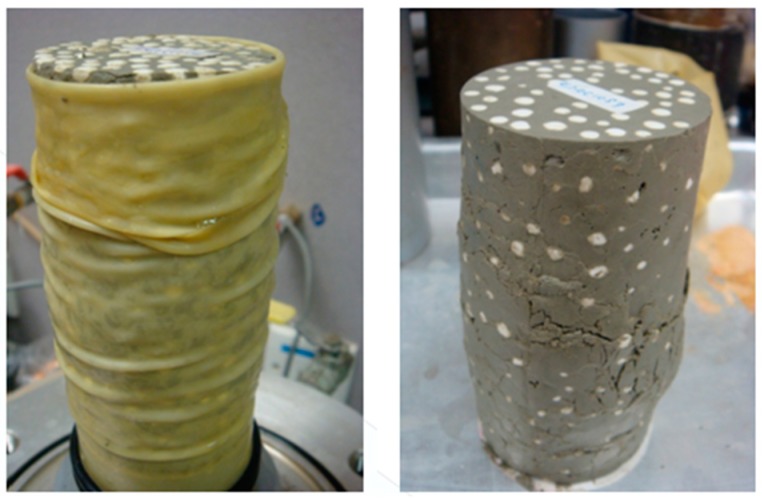
Elastoplastic failure for the higher E/S ratio.

A series of stress–strain curves of the EPS-cement mixed lightweight clay specimens were obtained by the UU tests in laboratory. The relationships between axial stress and axial strain of the EPS-cement lightweight clay specimens after seven curing days are shown in Figure 7 for cement ratio 10% and in Figure 8 for cement ratio 15%. It is clearly seen that both E/S ratio and confining pressure had significant effects on compressive strength and stress–strain behaviors. For a particular C/S ratio and a curing period, the compressive strength increased with the increase of confining pressure, but decreased with the increase of E/S ratio. When confining pressure was zero and the E/S ratio was low, each stress–strain curve had an obvious peak stress. When confining pressure was larger than 50 kPa and E/S was not equal to 0%, the stress–strain curve in the UU triaxial tests did not have an ultimate stress. With the increase of E/S ratio, the lightweight clay specimen failed from shear failure to elastoplastic failure under higher confining pressure. The shear failure in Figure 9 was observed for the unconfined specimens (0 kPa) and the specimens with lower E/S ratio (for example EPS = 0%) but high cement content. The elastoplastic failure in Figure 10 was observed for specimens with high confining pressure and high E/S ratio. The specimen deforms uniaxially along the maximum principal stress axis without an apparent shear surface. EPS beads in lightweight clay have high compressibility and thus increase the plasticity of specimens. Therefore, the failure patterns of this lightweight clay depend on both confining pressure and E/S ratio. Further, increasing cement content can enhance the compressive strength of the EPS-cement lightweight clay.

### 3.3. Compressive Strength Versus Failure Strain 

**Figure 11:**
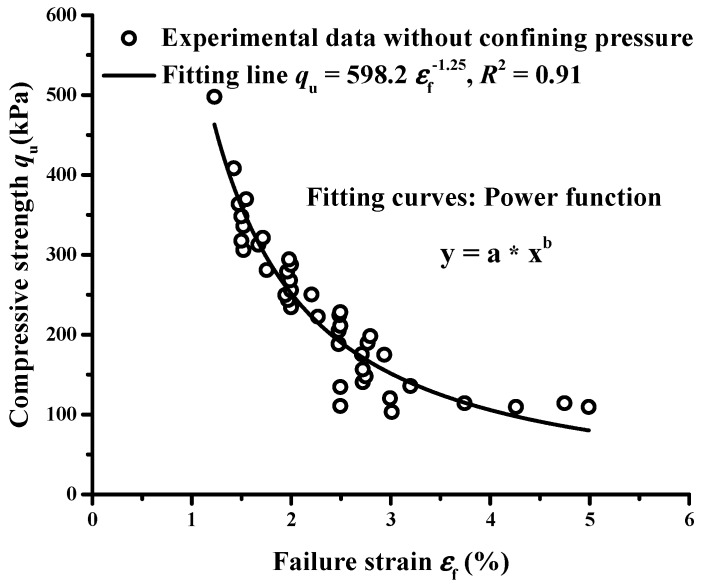
Relationship between compressive strength and failure strain without confining pressure.

The relationship between compressive strength $q_{u}$ and failure strain $\varepsilon_{f}$ without confining pressure is presented in Figure 11. The failure strain $\varepsilon_{f}$ (%) was in the range of 1.3% to 5% and had an inverse relationship with uniaxial strength $q_{u}$ (kPa). A power function $q_{u}=598.2\varepsilon_{f}^{-1.25}$ (kPa) was fitted with the correlation coefficient $R^{2}$ of 0.91. This fitting curve is consistent with those reported by Wang et al. [11] for carbonated reactive MgO-fly ash solidified sludge and by Du et al. [50] for the cement treated zinc-contaminated clay. Therefore, a power function can be used to characterize the relationship between $q_{u}$ and $\varepsilon_{f}$ of the EPS-cement lightweight clay.

### 3.4. Compressive Strength Versus Mass Density

**Figure 12:**
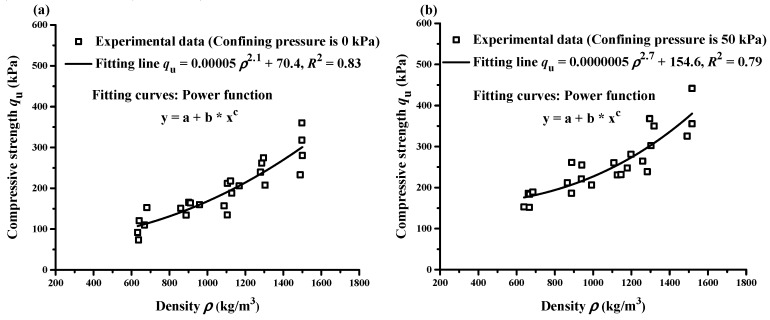

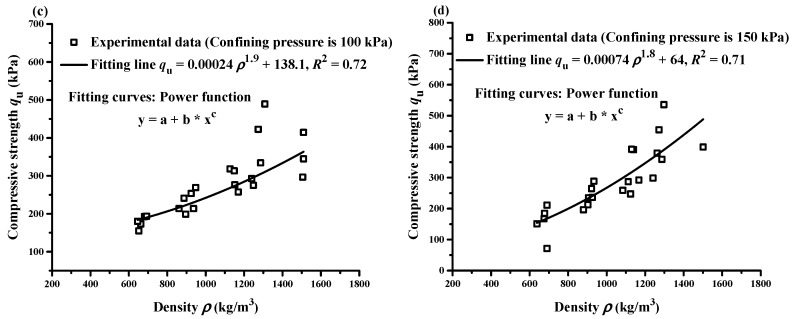
Relationship between compressive strength and density under different confining pressures: (**a**) 0 kPa; (**b**) 50 kPa; (**c**) 100 kPa and (**d**) 150 kPa.

The effects of mass density ρ on the compressive strength $q_{u}$ of specimens are shown in Figure 12 under different confining pressures. The compressive strength of lightweight clay increases approximately linearly with the increase of mass density. This is because lower mass density means higher volume of EPS beads and lower cement content in the lightweight clay. The effect of cement consolidation on the lightweight clay is weakened. The correlation between compressive strength $q_{u}$ and mass density ρ is best fitted with following power function as

(1) $$q_{u}=a_{1}+b_{1}\rho^{c_{1}}$$

where $a_{1}$, $b_{1}$, and $c_{1}$ are fitting parameters, $q_{u}$ is in kPa and ρ is in kg/m^3^.

The fitting functions under different confining pressures (0 kPa, 50 kPa, 100 kPa and 150 kPa) are shown in Figure 12a–d. Their corresponding correlation coefficients $R^{2}$ are 0.83, 0.79, 0.72 and 0.71, respectively. This power function is of significance to determine or check the compressive strength based on the mass density of EPS-cement lightweight clay in building construction and geotechnical engineering.

### 3.5. Compressive Strength Versus Curing Time

**Figure 13:**
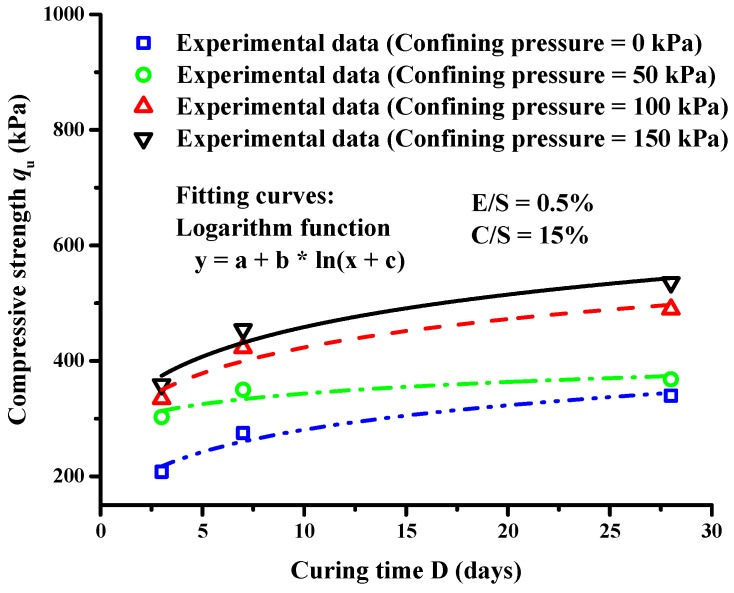
Compressive strength development of lightweight cement clay with curing time under different confining pressures.

Figure 13 shows the effect of curing time on the compressive strength of lightweight clay under different confining pressures, the E/S ratio of 0.5%, and the C/S ratio of 15%. With the increases of curing time, the compressive strength of lightweight clay under different confining pressures increased in the form of a logarithmic function. The compressive strength $q_{u}$ of lightweight clay without confining pressure was 207.7 kPa and 339.5 kPa after three and 28 curing days, respectively. The compressive strength increased by 64% from three to 28 curing days. For other confining pressures of 50 kPa, 100 kPa and 150 kPa, the compressive strength increased by 22%, 47% and 50%, respectively. The relationship between compression strength $q_{u}$ and curing time D can be expressed by:

(2) $$q_{u}=a_{2}+b_{2}\ln(D+c_{2})$$

where $a_{2}$, $b_{2}$ and $c_{2}$ are the fitting parameters.

The fitting formulae and correlation coefficients $R^{2}$ under five different confining pressures are listed in Table 3. It can be seen that this logarithmic function can well describe the relationship between $q_{u}$ and D under these confining pressures. The compressive strength was 340 kPa and 536 kPa under the confining pressure of 0 kPa and 150 kPa, respectively, which was increased by 58% after 28 curing days. Therefore, both confining pressure and curing time have important impacts on the compressive strength of lightweight clay.

## 4. Conclusions

A series of UU triaxial tests were conducted to investigate the physical and mechanical properties of the EPS-cement lightweight clay, such as the mass density, stress–strain behaviors, the relationship between compressive strength and failure, mass density and curing time. From these results, following conclusions can be drawn:

Firstly, the EPS beads had much lower unit weight and the E/S ratio was a key factor to control the mass density of EPS-cement lightweight clay. The mass density of EPS-cement lightweight clay decreased with the increase of E/S ratio. The E/S ratio increased from 0% to 4%, the mass density of EPS-cement lightweight clay after three curing days decreased by 55.6% for the C/S ratio 10% and 54.9% for the C/S ratio 15%, respectively.

Secondly, increasing cement content could enhance the compressive strength, and the E/S ratio and confining pressure determined the failure patterns of the EPS-cement lightweight clay. The shear failure occurred in the lightweight clay specimen without a confining pressure and lower E/S ratio. EPS beads were highly compressible and thus increased the plasticity of specimens. The shear failure changed to the elastoplastic failure with the increase of E/S ratio and confining pressure of the lightweight clay.

Thirdly, the relationships of the compressive strength $q_{u}$ with failure strain $\varepsilon_{f}$ and mass density ρ of EPS-cement lightweight clay could be described by power functions. High compressive strength $q_{u}$ corresponded to smaller failure strain $\varepsilon_{f}$ and the relationship in this study was $q_{u}=598.2\varepsilon_{f}^{-1.25}$ (kPa) with $R^{2}=0.91$. Higher mass density means more cement and lower EPS beads content of specimens and higher compressive strength.

Finally, both curing time and confining pressure were important to compressive strength. A logarithmic function can describe the relationship between compressive strength $q_{u}$ and curing time D under five different confining pressures. The compressive strength increased by 64%, 22%, 47% and 50% for five different confining pressures (0 kPa, 50 kPa, 100 kPa and 150 kPa), respectively from three to 28 curing days.

## Figures and Tables

**Table 1:** Properties of Singapore marine clay.

Property	Value
Original water content (%)	54.19
Initial void ratio	1.29
Specific gravity	2.65
Bulk density (g/cm^3^)	1.61
Liquid limit (%)	87.75
Plasticity limit (%)	40.04
Plasticity index (%)	47.71
Shear strength (kPa)	18.6–24.5

**Table 2:** Component ratios to dry clay by mass in EPS-cement lightweight clay.

Water to Clay, W/S (%)	Cement to Clay, C/S (%)	EPS to Clay, E/S (%)
100	10	0.0, 0.5, 1.0, 2.0, 4.0
100	15	0.0, 0.5, 1.0, 2.0, 4.0

**Table 3:** Fittings of compressive strength and curing time under different confining pressures.

Confining Pressure (kPa)	Fitting Equation	$R^{2}$
0	$q_{u}=115.7+67.6\ln(D+1.5)$	0.97
50	$q_{u}=265.1+32.1\ln(D+1.5)$	0.83
100	$q_{u}=231.7+78.5\ln(D+1.5)$	0.94
150	$q_{u}=239.0+89.9\ln(D+1.5)$	0.95
